# Evaluation of Real‐Time Cardiovascular Flow MRI Using Compressed Sensing in a Phantom and in Patients With Valvular Disease or Arrhythmia

**DOI:** 10.1002/jmri.29702

**Published:** 2025-01-20

**Authors:** Tania Lala, Lea Christierson, Petter Frieberg, Daniel Giese, Peter Kellman, Nina Hakacova, Pia Sjöberg, Ellen Ostenfeld, Johannes Töger

**Affiliations:** ^1^ Clinical Physiology, Department of Clinical Sciences Lund, Lund University Skåne University Hospital Lund Sweden; ^2^ Biomedical Engineering Lund University Lund Sweden; ^3^ Pediatric Heart Centre, Department of Clinical Sciences Lund, Lund University Skåne University Hospital Lund Sweden; ^4^ Magnetic Resonance Siemens Healthineers AG Erlangen Germany; ^5^ National Heart, Lung, and Blood Institute Bethesda MD USA

**Keywords:** real‐time, blood flow, online compressed sensing, arrhythmia, phantom, in‐vivo

## Abstract

**Background:**

Real‐time (RT) phase contrast (PC) flow MRI can potentially be used to measure blood flow in arrhythmic patients. Undersampled RT PC has been combined with online compressed sensing (CS) reconstruction (CS RT) enabling clinical use. However, CS RT flow has not been validated in a clinical setting.

**Purpose:**

Evaluate CS RT in phantom and patients.

**Study Type:**

Prospective.

**Population:**

Flow phantom (60 cycles/min: N = 10, 120 cycles/min: N = 12), sinus rhythm patients, no regurgitation (N = 20) or suspected aortic regurgitation (N = 10), arrhythmia patients (N = 10).

**Field Strength/Sequence:**

1.5 T, 2D gated PC, CS RT PC, RT cine with arrhythmia rejection.

**Assessment:**

Phantom experiments tested the accuracy of CS RT cardiac output and peak flow rate at 60 and 120 cycles/min against gated PC. For sinus rhythm patients, cardiac output, peak flow rate, and regurgitation fraction in the ascending aorta and/or pulmonary artery were evaluated against gated PC. Cardiac output in patients with arrythmia was evaluated against RT cine with arrhythmia rejection.

**Statistical Tests:**

Bland Altman, correlation, Mann–Whitney test, Wilcoxon signed‐rank test.

**Results:**

Cardiac output bias ± SD for CS RT in the phantom was −0.0 ± 0.2 L/min (0.5 ± 3%, *P* = 0.76) at 60 cycles/min and 0.2 ± 0.3 L/min (4 ± 4%, *P* = 0.0016) at 120 cycles/min. Correspondingly, peak flow rate bias was −23 ± 6 mL/s (−7 ± 2%, *P* < 0.0001) and −73 ± 25 mL/s (−23 ± 4%, *P* < 0.0001). In patients, regurgitant fraction was −4 ± 0.5% (−23 ± 4%, *P* = 0.0025). Cardiac output bias in patients in sinus rhythm was −0.1 ± 0.5 L/min (−2 ± 10%, *P* = 0.99) (with regurgitation) and −0.3 ± 0.6 L/min (−5 ± 11%, *P* = 0.035) (without regurgitation). Peak flow rate bias was −60 ± 31 mL/s (−13 ± 6%, *P* < 0.0001) (with regurgitation) and −64 ± 32 mL/s (−16 ± 8%, *P* < 0.0001) (without regurgitation). Cardiac output bias was −0.4 ± 0.6 L/min (−9 ± 11%, *P* < 0.003) in arrhythmia patients.

**Data Conclusions:**

CS RT flow could potentially serve as a clinical tool for patients with or without valvular disease or arrhythmia, with accurate cardiac output and regurgitation fraction quantification.

**Plain Language Summary:**

Accurate flow assessment is important in clinical evaluation of cardiac patients, but in the presence of irregular heart rhythm flow assessment is challenging. We have evaluated a new method using cardiac magnetic resonance imaging and real‐time flow for blood flow assessment in cardiac patients. The method was tested against a reference method in a phantom flow model in low and high heart rates, and in cardiac patients with and without irregular heart rhythm and in different vessels. We found the cardiac magnetic resonance imaging real time flow method accurate and therefore promising for clinical implementation.

**Evidence Level:**

1

**Technical Efficacy:**

Stage 1

Cardiovascular blood flow quantification with high accuracy and precision is important for diagnosis and treatment of disease.^1^ Cardiac‐gated, two‐dimensional phase contrast (PC) MRI allows noninvasive blood flow measurement.^2^, ^3^ Gated PC has been thoroughly validated since first described in the 1980s^4^, ^5^, ^6^ and is considered a standard clinical tool for blood flow assessment, even in the presence of regurgitant blood flow as in valvular disease.^1^, ^7^ However, gated PC is often not suitable for imaging in arrhythmia and could result in reduced image quality and quantification errors.

Real‐time (RT) PC permits fast imaging of blood flow without cardiac gating,^8^, ^9^ making RT feasible for imaging in arrhythmia.^10^, ^11^, ^12^ However, RT suffers from low spatial and temporal resolution, which may limit accuracy and precision.^13^

Multiple approaches have been proposed to improve spatiotemporal resolution of RT flow with undersampled data and advanced image reconstruction. These include: 1) compressed sensing (CS) combined with non‐cartesian trajectories, for example, radial^14^, ^15^, ^16^ and spiral,^17^ 2) parallel imaging with low‐rank modeling,^12^ and 3) deep learning.^18^ These approaches typically require image reconstruction after scanning (*off‐line*), hindering clinical implementation.

Recently, a research sequence with *online* CS reconstruction was developed and validated in a phantom and healthy volunteers.^19^ Reconstruction is performed at the scanner and could facilitate clinical integration. However, the framework has not been validated in patients.

Therefore, the aims of this study were to: 1) validate the sequence in a flow phantom with simulated high heart rates as seen in patients, 2) extend the validation to cardiac patients in sinus rhythm, with and without valvular regurgitation, against the established gated PC method, and 3) perform validation in patients with arrhythmia.

## Materials and Methods

This study included validation experiments in vitro (phantom) and in vivo (patients). The study was approved by the Regional Ethical Review Board in Lund, Sweden and complied with the Helsinki declaration. Participants were recruited prospectively and provided signed written informed consent. Scans were performed using a 1.5 T MRI scanner with a 32‐channel spine coil and an 18‐channel anterior coil (MAGNETOM Sola, Siemens Healthineers, Forchheim, Germany). Coil elements used were automatically selected by the scanner.

### In Vitro Experiments

#### DESCRIPTION OF FLOW PHANTOM

**FIGURE 1:**
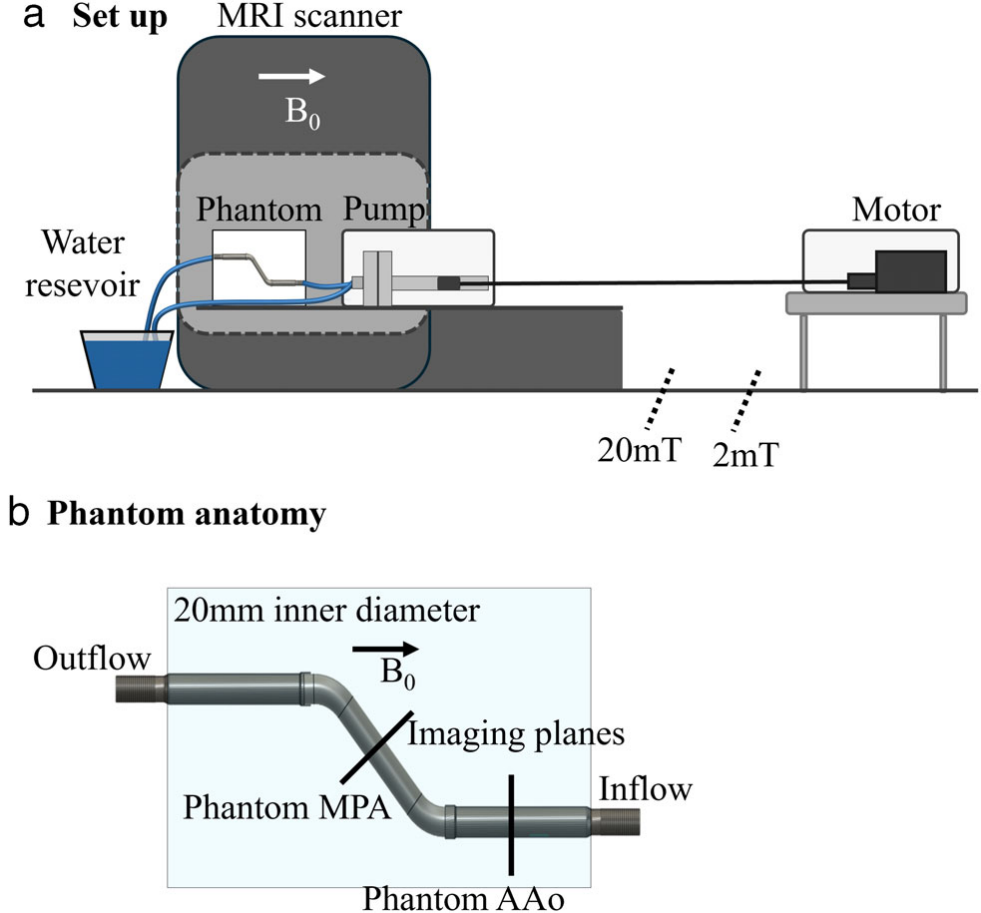
Description of flow phantom experiments, (**a**) setup at the scanner room, (**b**) flow phantom anatomy. AAo = ascending aorta; MPA = pulmonary artery.

The experimental setup included a flow phantom, a pulsatile pump, and a water reservoir (Fig. 1a).

The flow phantom (Fig. 1b) was designed and 3D printed in‐house. It consisted of a plastic tube (20 mm inner diameter, 1 mm wall thickness) in two sections: 1) in the feet‐head direction of the scanner mimicking the ascending aorta (AAo) and 2) at a 45° angulation to simulate slice positioning in the pulmonary artery (MPA). The tube was fixed in a box filled with water. Description of experimental equipment can be found in the Supporting Information.

#### PHANTOM EXPERIMENTS

Eleven different pump settings were used, set to approximate cardiac output in patients (3–11 L/min). Five were generated with 60 cycles per minute and the remaining six with 120 cycles per minute. Imaging was performed in: 1) transverse to the phantom AAo and 2) transverse to the phantom MPA. The isocenter was located between the two imaging planes. In total, 22 experiments (11 flows × 2 planes) were performed. The phantom was placed at the isocenter and the table remained fixed.

The MRI protocol included gated two‐dimensional PC as reference, and the research CS RT sequence. Timer and beaker was performed before and after each MRI experiment (approximately 6 minutes apart) as in a previous study.^20^ The two timer and beaker flow measurements were averaged and compared to cardiac output from gated PC to assess accuracy of the reference method. CS RT data was acquired at the same positions and time points as gated PC.

### In Vivo Experiments

#### STUDY COHORT

In vivo validation was performed with 40 cardiac patients clinically referred for MRI at Skåne University Hospital, Lund, Sweden. The inclusion groups were: 1) sinus rhythm without regurgitation (N = 20), 2) sinus rhythm with suspected aortic regurgitation (N = 10), and 3) patients with arrhythmia (N = 10). Patients were recruited from the clinical workflow at our department from November of 2022 to November of 2023.

In patients without regurgitation, imaging was performed in AAo (N = 10) or MPA (N = 10). Patients with suspected aortic regurgitation underwent imaging only in the AAo.

All patients with arrhythmia had flow scans in the AAo, and when the clinical question included MPA flow, CS RT in the MPA was added. The arrhythmia types included were atrial fibrillation (N = 3), frequent ventricular extra beats (N = 5), frequent supraventricular extra beats (N = 2).

### Flow MRI Sequences

Table 1 shows flow sequence parameters. A product gated two‐dimensional PC sequence without acceleration was used as reference. The sequence uses segmentation factor one, i.e., acquired temporal resolution 9.8 msec, which results in a 3‐minute scan. The acquisition is done without respiratory gating and data is averaged over the multiple respiratory cycles, reducing respiratory artifacts. Image reconstruction creates 35 timeframes, as a reasonable dataset to analyze.

**TABLE 1:** Flow Pulse Sequence Parameters

Parameter	Gated	Real‐Time	
Acceleration	None	CS (*R* = 7.9)	CS (*R* = 13.3)
Shared velocity encoding	No	Yes	No
TR (msec)/TE (msec)/Flip angle (°)	4.9/2.67/20	3.42/2.43/12	3.42/2.43/12
Field of view (read × phase × slice mm^3^)	400 × 323 × 5	380 × 308 × 6	380 × 308 × 6
Spatial resolution acquired (read × phase × slice mm^3^)	1.92 × 1.92 × 5	2.96 × 2.8 × 6	2.3 × 2.85 × 6
Spatial resolution reconstructed (read × phase × slice mm^3^)	1.92 × 1.92 × 5	1.18 × 1.18 × 6	1.18 × 1.18 × 6
Temporal resolution acquired (msec)	9.8	95.8	54.7
Temporal resolution reconstructed (msec)	28.5 (for 60 bpm) 14.2 (for 120 bpm)	47.9	54.7
Number of reconstructed timeframes	35	207	182
VENC (cm/s)	200	200	200
Bandwidth (Hz/pixel)	454	781	781
Scan time (s)	180	10	10

CS = compressed sensing; TR = repetition time; TE = echo time; VENC = velocity encoding.

CS RT was an undersampled, two‐dimensional PC sequence with sparse incoherent Cartesian sampling and CS reconstruction.^19^ The acquisition window was set to 10 seconds to include multiple heartbeats in the analysis and reduce respiratory effects, keep the number of reconstructed timeframes low and the scan time fast. The acquisition time was fixed and not varied with respiratory rate to simplify application of CS RT.

Two CS acceleration factors were tested, *R* = 7.9 and *R* = 13.3. Acceleration factor *R* = 13.3 had acquired and reconstructed temporal resolution of 54.7 msec. Acceleration factor *R* = 7.9 had an acquired temporal resolution of 95.8 msec and was combined with shared velocity encoding (SVE) for a reconstructed temporal resolution of 47.9 msec. Shared velocity encoding doubled the reconstructed temporal resolution by sharing images acquired with different bipolar gradient polarities between consecutive timeframes.^21^ Protocols were designed to maximize spatial resolution with temporal resolution around 50 msec. The CS reconstruction was performed online.

The imaging plane was placed perpendicular to the main flow direction and was identical for all three PC MRI acquisitions.

### RT Cine With Arrhythmia Rejection

In arrhythmia patients, CS RT cardiac output was assessed and evaluated against an RT balanced steady‐state free precession (bSSFP) cine sequence with retrospective arrhythmia rejection. The imaging pipeline encompassed a real‐time cine acquisition with 18 slices in the short axis view covering the ventricles. The acquired data were retrospectively reconstructed into one cardiac cycle employing arrhythmia rejection and motion correction. Each slice acquisition was 4 seconds long. RT cine was used for left ventricular cardiac output calculation in the 10 patients with arrhythmia. In four of these, pulmonary flow was also acquired and right ventricular cardiac output calculated.

The RT cine method was validated for cardiac output quantification against gated PC in patients in sinus rhythm (N = 9). Short axis stacks were acquired with RT cine and AAo flow data acquired with gated PC.

### Data Analysis

All image analysis was performed in Segment version 3.3 (Medviso AB, Lund, Sweden).^22^ The vessel region of interest (ROI) was delineated semi‐automatically^20^ followed by manual corrections when needed.

Cardiac output and peak flow rate were assessed in the phantom and patients with sinus rhythm, and only cardiac output was assessed in arrhythmia patients. The effect of heart rate on quantification error was examined in sinus rhythm patients without regurgitation by comparing patients with heart rate below and above the median heart rate. In patients with suspected aortic regurgitation, the CS RT protocols were investigated in whether they changed the regurgitation severity categorization compared to gated PC.^23^

Calculation of flow parameters from CS RT was done from multiple non‐truncated flow curves captured in the 10‐second acquisition. Cardiac output was calculated in L/min by assessing the total flow volume and dividing by the total time for the selected heartbeats. This methodology was followed for sinus rhythm and arrhythmia. No arrhythmia rejection was employed. In patients with sinus rhythm peak flow rate was calculated as the average of all flow rate peaks in the flow curves used for cardiac output calculation. Cardiac output was the only flow parameter validated in patients with arrhythmia due to lack of reference method for peak flow rate.

Gated PC cardiac output was computed as the product of stroke volume and heart rate. Regurgitation fraction was quantified as the ratio of backward and forward flow in CS RT and gated PC.

Background phase correction was performed in all PC images. Static regions were manually delineated and used for the calculation of a linear fit that was subtracted from the image. In the phantom, the static regions included the static water surrounding the tube, while in vivo they included the chest and back. The mean value of the linear fit in the vessel ROI was calculated in the phantom 60 cycle/minute experiments (AAo: N = 5, MPA: N = 5) and in 10 patients in sinus rhythm and no valve disease (AAo: N = 5, MPA: N = 5).

RT cine cardiac output was computed from the volumetric difference in end diastole (EDV) and end systole (ESV) multiplied by the heart rate.

### Interobserver Variability

All delineations were performed by two observers. All CS RT images were delineated by TL (4 years of experience in flow MRI) and JT (16 years of experience in flow MRI). Ventricular delineations with RT cine in arrhythmia and sinus rhythm were performed by PS (11 years of experience in flow MRI) and EO (European Association of Cardiovascular Imaging [EACVI] level 3, 18 years of experience in flow MRI).

### Statistical Analysis

Statistical analyses were performed in GraphPad Prism version 9.4.1 (GraphPad Software, San Diego, California, USA). Correlation plots with Pearson's correlation coefficient (*r*) and modified Bland–Altman analyses with bias ± SD in the text and limits of agreement (LOA; 1.96 × SD) in the figures were used to compare methods, to compare flow data before and after each MRI experiment in the phantom and for interobserver variability analyses. Unpaired Mann–Whitney two‐sided *U*‐tests were used to examine 1) if the heart rate affected measurements in sinus rhythm patients without regurgitation, 2) if CS RT performed differently in patients with or without valvular disease, and 3) if background velocity in the phantom and patients is significantly different between sequences. Wilcoxon's signed‐rank test was used to test whether errors were significantly different from zero.

## Results

Online CS image reconstruction was completed within 10 seconds after acquisition for both CS RT protocols. Table 2 shows patient characteristics. *R*‐values and absolute and relative biases ± SD in flow quantification between CS RT and references are presented throughout this section and in Table 3. The median number of coil elements used for flow scans was 20 (range: 10–38).

**TABLE 2:** Patient Characteristics

Patient Group	Sinus Rhythm, No Valvular Disease	Sinus Rhythm, Suspected Valvular Regurgitation	Arrhythmia, No Valvular Disease
Males/females (N)	8/12	8/2	8/2
Age (years)	55.5 ± 20.5	60.8 ± 16.4	58.2 ± 19.8
Body surface area (m^2^)	1.9 ± 0.2	1.9 ± 0.1	2.0 ± 0.2
Height (cm)	172 ± 10.5	176.5 ± 7	177.2 ± 7.5
Weight (kg)	76 ± 13.5	75.2 ± 8.4	82.4 ± 8.6
Stroke volume (mL)	85.9 ± 16.5	77.3 ± 17	91 ± 26.5
Forward flow (mL)	88 ± 16.5	101.3 ± 32	N/A
Regurgitant flow (mL)	2 ± 1.4	24.6 ± 22	N/A

Data expressed as absolute numbers or mean ± SD.

**TABLE 3:** Statistical Results

Parameter	Subjects	Vessel	N	Absolute Difference		Relative Difference (%)		*r*	
				*R* = 7.9, SVE	*R* = 13.3	*R* = 7.9, SVE	*R* = 13.3	*R* = 7.9, SVE	*R* = 13.3
Cardiac output (L/min)	Phantom	AAo 60 bpm	5	0.0 ± 0.2	−0.1 ± 0.0	0.8 ± 2.7	−1.9 ± 1	0.98	1.00
		AAo 120 bpm	6	0.2 ± 0.3	−0.0 ± 0.4	3.8 ± 3.5	0.3 ± 4.3	1.00	0.99
		MPA 60 bpm	5	0.1 ± 0.2	0.1 ± 0.2	1.7 ± 3.7	1.3 ± 3.5	0.98	0.99
		MPA 120 bpm	6	0.4 ± 0.2	0.3 ± 0.2	6.3 ± 3.8	3.9 ± 3.0	0.99	1.00
	Sinus, no regurg	AAo	10	−0.2 ± 0.5	−0.2 ± 0.6	−4.1 ± 8.8	−2.6 ± 10.8	0.96	0.94
		MPA	10	−0.5 ± 0.7	−0.3 ± 0.7	−7.3 ± 11.3	−5.8 ± 12.9	0.93	0.93
	Sinus, regurg	AAo	10	−0.1 ± 0.5	−0.0 ± 0.5	−2.7 ± 11.1	−0.6 ± 11.2	0.88	0.85
	Arrhythmia	AAo + MPA	10 (10 AAo, 4 MPA)	−0.38 ± 0.6	−0.44 ± 0.6	−8.5 ± 12.4	−8.4 ± 11.1	0.90	0.86
Peak flow rate (mL/s)	Phantom	AAo 60 bpm	5	−25.2 ± 4.2	−17.5 ± 5.7	−7.6 ± 1.5	−5.3 ± 2.1	1.00	1.00
		AAo 120 bpm	6	−83.5 ± 30.6	−75.8 ± 29.3	−25.5 ± 2.3	−23.1 ± 4.5	1.00	0.99
		MPA 60 bpm	5	−26.9 ± 5.9	−22 ± 6	−8.7 ± 1.2	−7.0 ± 0.9	1.00	1.00
		MPA 120 bpm	6	−70.3 ± 20.7	−60.6 ± 20.3	−23.8 ± 3.4	−20.2 ± 4.2	0.99	0.99
	Sinus, no regurg	AAo	10	−74.3 ± 28.4	−60.2 ± 31.7	−18.3 ± 5.6	−14.4 ± 6.2	0.98	0.97
		MPA	10	−68.6 ± 33.9	−53.5 ± 36.0	−18.3 ± 10.3	−14.1 ± 10.8	0.95	0.95
	Sinus, regurg	AAo	10	−84.0 ± 32.4	−48.8 ± 23.6	−16.3 ± 6.3	−9.5 ± 5.0	0.98	0.99
Regurgitation fraction (%)	Sinus, regurg	AAo	10	−4.5 ± 4.9	−2.3 ± 4.0	−38.8 ± 40.3	−31.4 ± 57.4	0.94	0.96

Bland–Altman and correlation analysis of flow measurements in the phantom and patients between compressed sensing phase contrast real‐time (CS RT) MRI vs. gated phase contrast MRI as a reference. Data expressed as mean ± SD. AAo = ascending aorta; MPA = pulmonary artery; Regurg = regurgitation; SVE = shared velocity encoding; *r* = Pearson correlation coefficient.

### In Vitro

#### PHANTOM SETUP ACCURACY

Timer and beaker measurements before and after each MRI experiment showed cardiac output difference (cardiac output before‐after MRI) less than 2.5% in all cases, with a difference of −0.03 ± 0.09 L/min (−0.4 ± 1%).

Validation of reference gated PC against timer and beaker showed an overall bias of −0.02 ± 0.37 L/min (−0.3 ± 5%). The cardiac output bias in the phantom AAo imaging plane was 0.27 ± 0.20 L/min (4 ± 3%) and −0.31 ± 0.25 L/min (−5 ± 3%) for the phantom MPA plane.

#### CS RT IN THE PHANTOM

**FIGURE 2:**
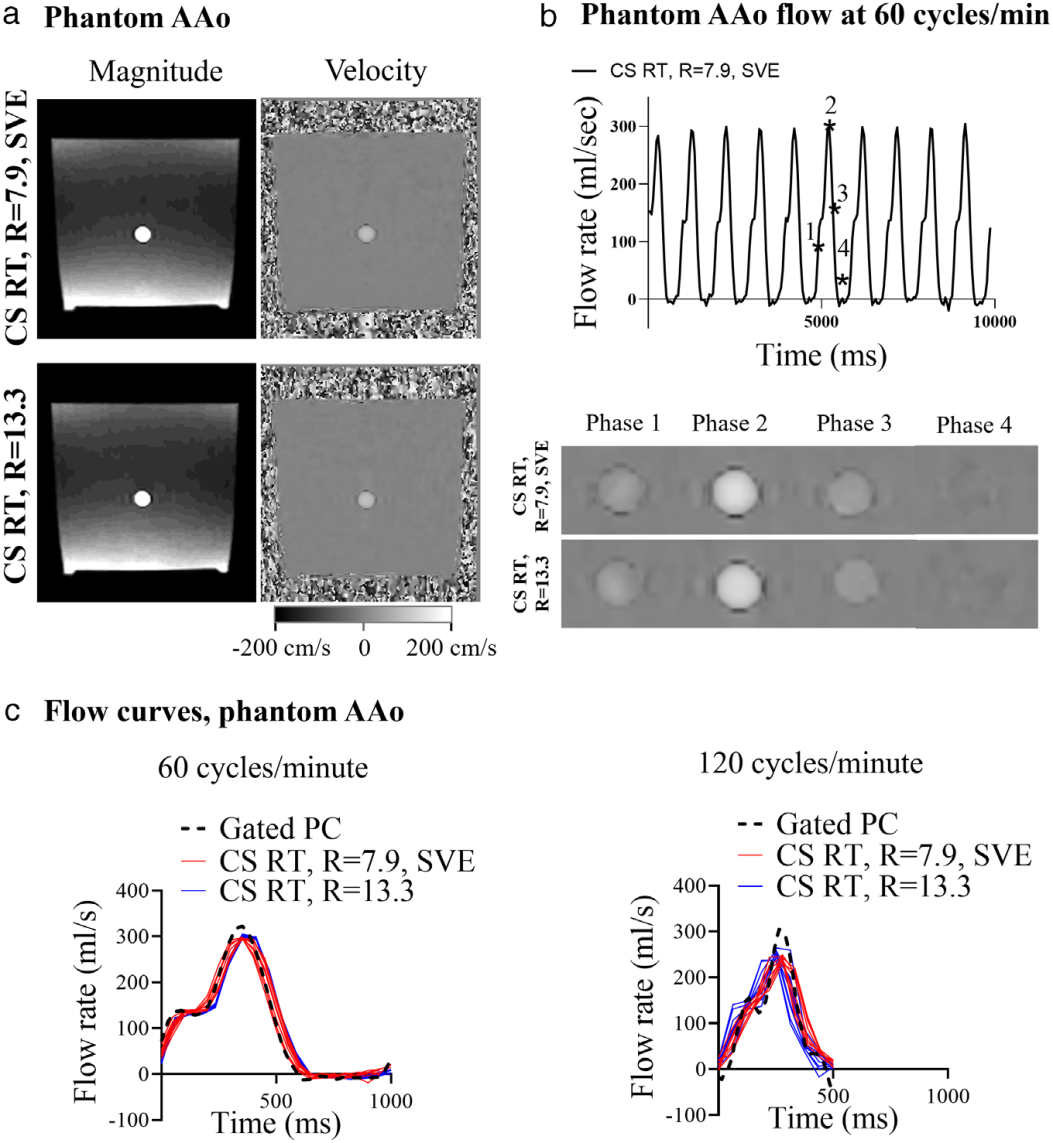
Phantom experiment results, (**a**) example MRI images in the phantom AAo imaging plane, as acquired with the CS RT, *R* = 7.9, SVE (top panels) and the CS RT, *R* = 13.3 (bottom panels), (**b**) the flow curve generated by the pump in the phantom AAo time as acquired with the CS RT, *R* = 7.9, SVE (top panel) protocol. Bottom panel shows zoomed flow images from selected phases. (**c**) Comparison of the overlapping heartbeats acquired with CS RT with the corresponding gated PC reference in the 60 cycles/minute experiments (left panel) and 120 cycles/minute experiments (right panel) in phantom AAo. AAo = ascending aorta; CS = compressed sensing; RT = real‐time; SVE = shared velocity encoding; PC = phase contrast.

Example CS RT images from the phantom AAo plane at peak systole are shown in Fig. 2a, while Fig. 2b shows flow over time (top) and zoomed flow images in four cardiac phases (bottom). Figure 2c shows flow curves in each CS RT cycle compared to the reference in the phantom AAo plane.

**FIGURE 3:**
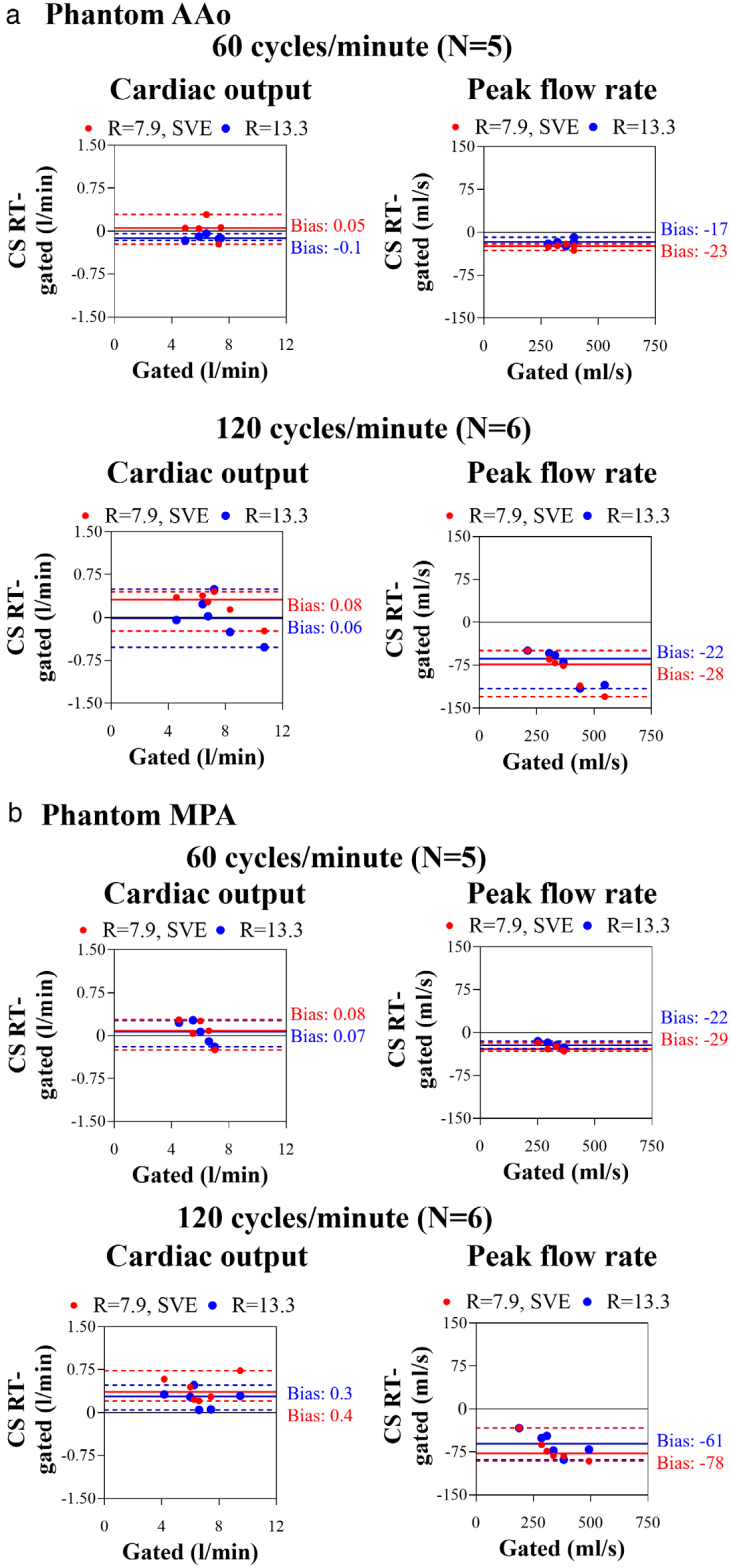
Bland–Altman analysis plots of cardiac output and peak flow measurements from compressed sensing real‐time phase contrast MRI (CS RT) using gated phase contrast MRI as a reference at (**a**) the phantom ascending aorta (AAo) and (**b**) pulmonary artery (MPA) planes for 60 cycles/minute and 120 cycles/minute. Full lines: absolute biases, dotted lines: limits of agreement, red: data acquired with CS RT, *R* = 7.9, SVE, blue: data acquired with CS RT, *R* = 13.3. AAo = ascending aorta; MPA = pulmonary artery; CS = compressed sensing; RT = real‐time; SVE = shared velocity encoding.

Flow quantification from the phantom AAo and MPA imaging planes resulted in *r* ≥ 0.98 for cardiac output and *r* ≥ 0.97 for peak flow rate compared to gated PC (Table 3; Fig. 3). Cardiac output bias for both imaging planes was 0.0 ± 0.2 L/min (0.5 ± 3%, *P* = 0.76) and 0.2 ± 0.3 L/min (4 ± 4%, *P* = 0.0016) for 60 cycles/minute and 120 cycles/minute, respectively. Peak flow rate showed an underestimation in both imaging planes at 60 cycles/minute with −23 ± 6 mL/s (−7 ± 2%, *P* < 0.0001) and at 120 cycles/minute with −73 ± 25 mL/s (−23 ± 4%, *P* < 0.0001). The two CS RT protocols resulted in biases that did not differ by more than 4% across parameters. Results for each imaging plane and each CS RT protocol separately can be found in Table 3. Background phase analysis can be found in the Supporting Information.

### In Vivo

#### CS RT IN SINUS RHYTHM AND NO VALVULAR DISEASE

**FIGURE 4:**
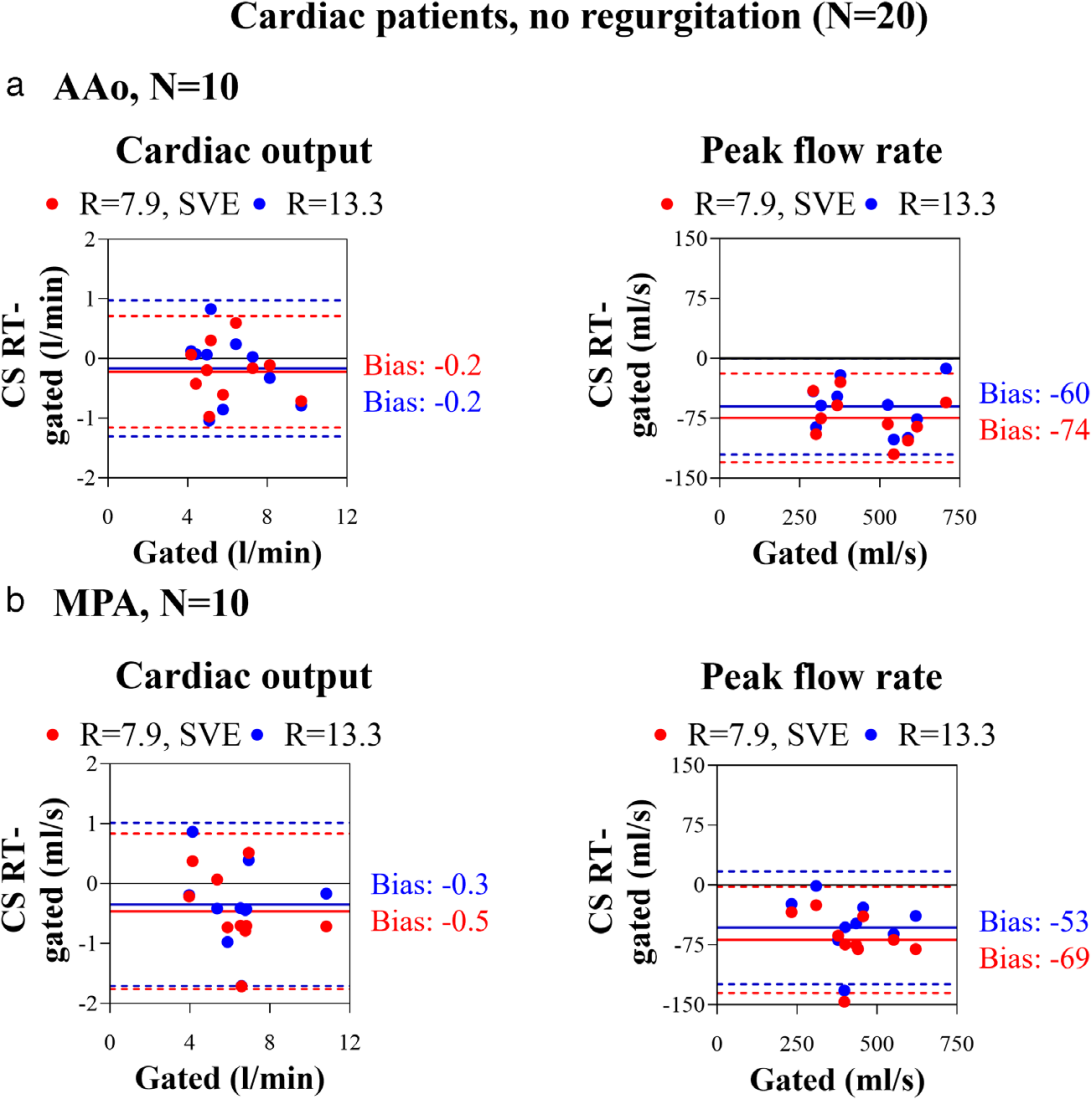
Bland–Altman analysis plots of cardiac output (top row) and peak flow rate (bottom row) measurements from compressed sensing real‐time phase contrast MRI (CS RT) using gated phase contrast MRI as a reference at (**a**) the ascending aorta (AAo) and (**b**) pulmonary artery (MPA) planes of cardiac patients without regurgitation. Full lines: absolute biases, dotted lines: 95% limits of agreement, red: data acquired with CS RT, *R* = 7.9, SVE, blue: data acquired with CS RT, *R* = 13.3. AAo = ascending aorta; MPA = pulmonary artery; CS = compressed sensing; RT = real‐time; SVE = shared velocity encoding.

In patients with sinus rhythm and no valvular disease, CS RT methods and gated PC correlated with *r* > 0.93 for all comparisons (cardiac output and peak flow rate of AAo and MPA) (Fig. 4). The absolute bias of cardiac output was −0.3 ± 0.6 L/min (−5 ± 11%, *P* = 0.0035), and of peak flow rate was −64 ± 32 mL/s (−16 ± 8%, *P* < 0.0001) for both CS sequences and in both AAo and MPA. Background phase analysis can be found in the Supporting Information.

#### EFFECT OF HEART RATE IN SINUS RHYTHM AND NO VALVULAR DISEASE

The mean heart rates during the gated and the two CS RT acquisitions ranged between 50 and 98 bpm, with a median of 68 bpm. Cardiac output bias was −0.1 ± 0.5 L/min (−2 ± 9%) in the patients with heart rate <68 bpm and −0.5 ± 0.6 L/min (−9 ± 12%) in patients with heart rate >68 bpm (*P* = 0.25). Peak flow rate bias was −59 ± 30 mL/s (−14 ± 6%) for heart rate <68 bpm, and −71 ± 35 mL/s (−19 ± 10%) >68 bpm (*P* = 0.46).

#### CS RT IN PATIENTS WITH SUSPECTED AORTIC REGURGITATION

**FIGURE 5:**
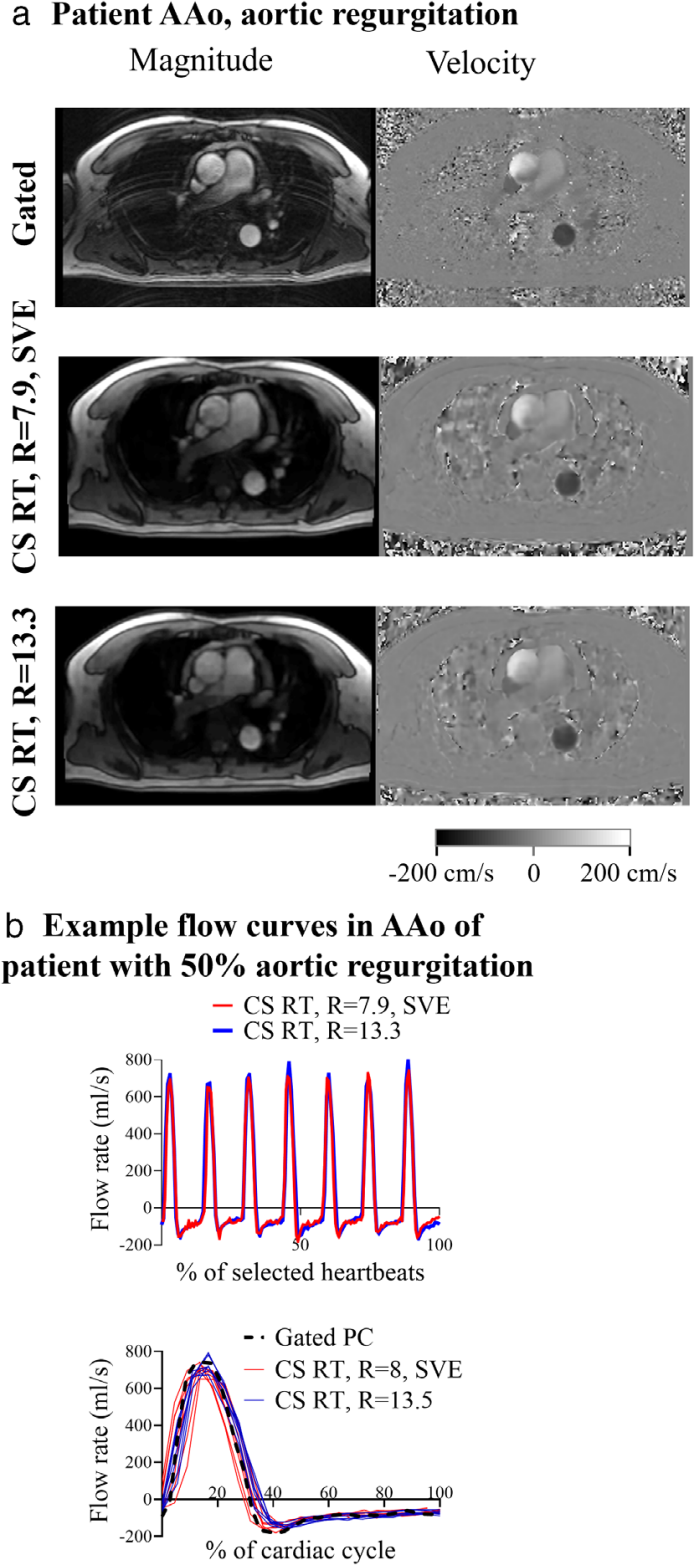
Results from an example patient with 50% aortic regurgitation. (**a**) MRI images at peak systole in the Aao, as acquired with compressed sensing real‐time phase contrast MRI (CS RT) with *R* = 7.9, SVE (top panel) and *R* = 13.3 (bottom panel), (**b**) example flow curves from the AAo, as acquired with CS RT (top panel) and after overlaying the consecutive heartbeats for comparison with the reference gated phase contrast (PC) sequence (bottom panel). AAo = ascending aorta; CS = compressed sensing; RT = real‐time; SVE = shared velocity encoding; PC = phase contrast.

**FIGURE 6:**
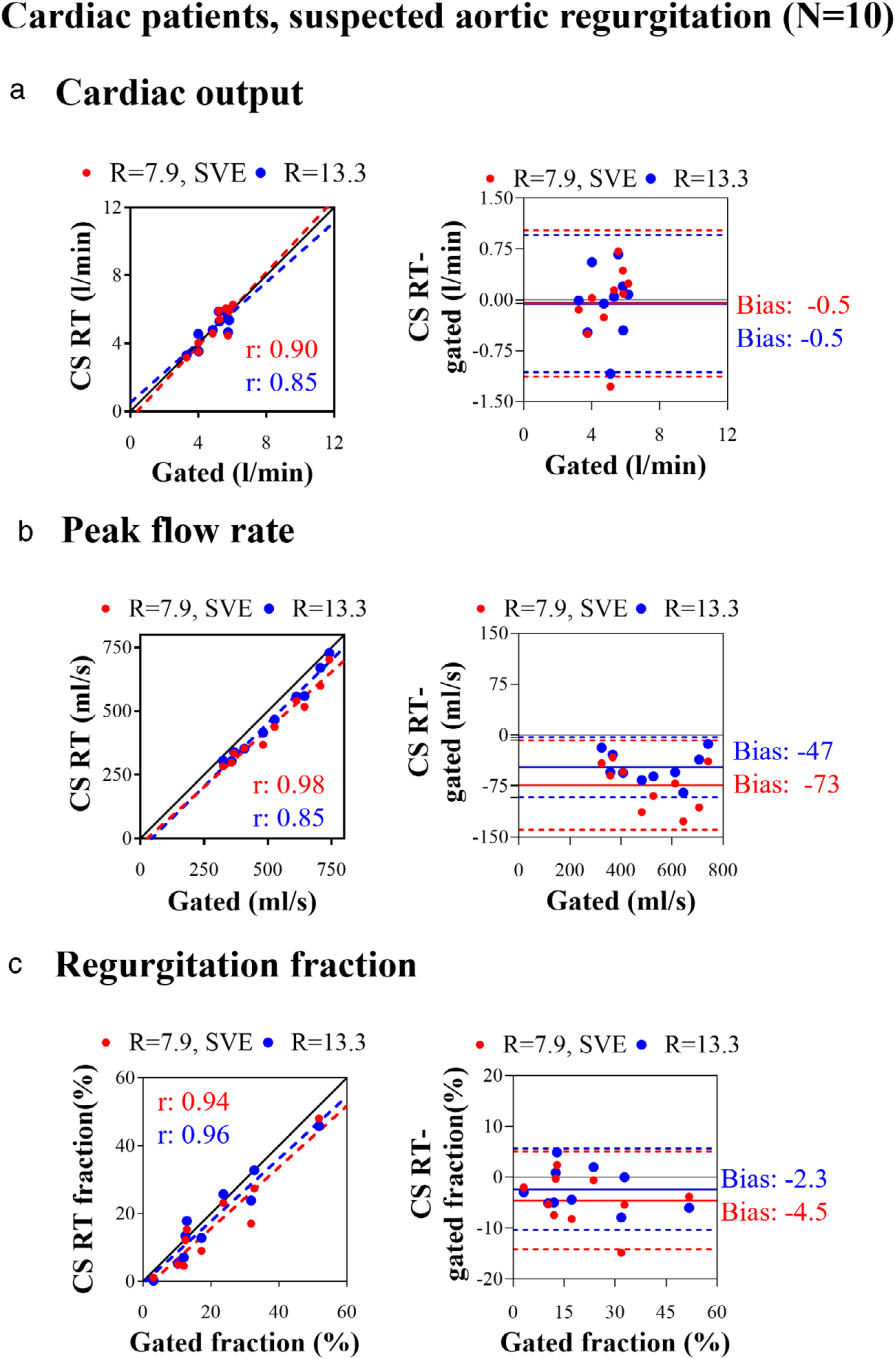
Correlation plots (left, full line: identity line, dotted lines: regression line) and Bland–Altman analysis plots (right panels, full lines: absolute biases, dotted lines: limits of agreement) of (**a**) cardiac output, (**b**) peak flow rate, and (**c**) regurgitation fraction measurements from CS RT and gated PC. Red: data acquired with CS RT, *R* = 7.9, SVE, blue: data acquired with CS RT, *R* = 13.3. AAo = ascending aorta; MPA = pulmonary artery; CS = compressed sensing; RT = real‐time; SVE = shared velocity encoding.

Example CS RT images acquired in a patient with aortic regurgitation are shown in peak systole in Fig. 5a. Figure 5b shows CS RT flow curves in the AAo from a patient with 50% aortic regurgitation. The top flow curve of Fig. 5b shows the CS RT result, while the bottom flow curve shows how the individual sequential CS RT heartbeats compare to the flow curve acquired by gated PC. The *r*‐values between CS RT methods and gated PC were >0.85 for all comparisons (Fig. 6). Bias of cardiac output was −0.1 ± 0.5 L/min (−2 ± 10%, *P* = 0.99), and was not statistically significant compared to measurements in the AAo of patients without valve disease (*R* = 7.9, SVE: *P* = 0.35, *R* = 13.3: *P* = 0.91). Peak flow rate bias was −60 ± 31 (−13 ± 6%, *P* < 0.0001), which was also not significantly different to measurements in the AAo of patients without valve disease (*R* = 7.9, SVE: *P* = 0.39, *R* = 13.3: *P* = 0.28). Regurgitation fraction absolute biases were −3.4 ± 2.5 (−35 ± 49%, *P* = 0.0025) (Table 3; Fig. 6c).

The CS RT measurements changed regurgitation severity class in two patients. In the first patient, the CS RT, *R* = 7.9, SVE changed the class of the patient. The numerical change was 5.4% which is within the interobserver variability error. In the second patient, both protocols changed the class of the patient with numerical changes of 15.1% and 7.9%, where only the latter could be explained by interobserver variability.

#### CS RT IN PATIENTS WITH ARRHYTHMIA

**FIGURE 7:**
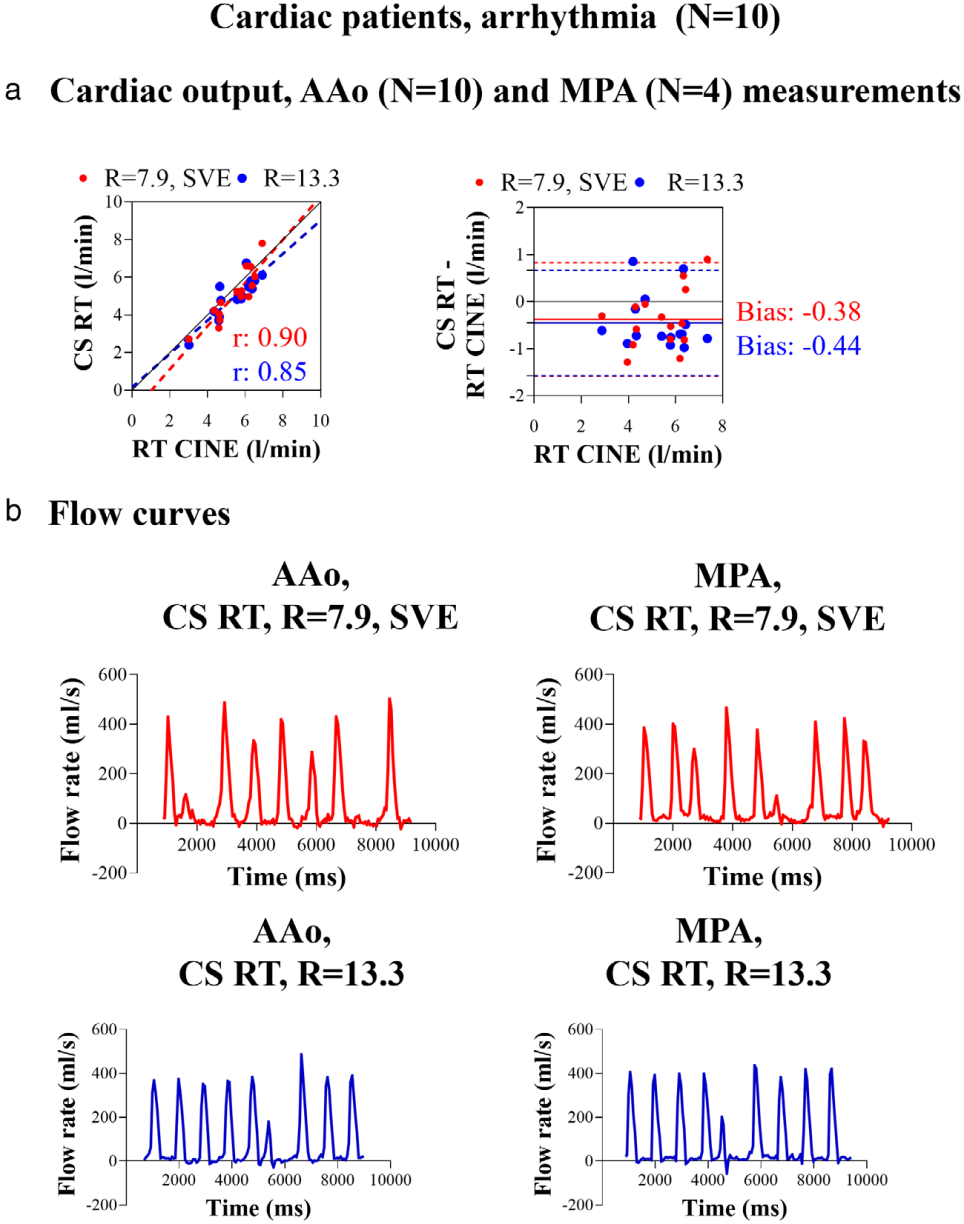
Results from patients with arrhythmia. Correlation (full line: identity line, dotted lines: regression line) and Bland–Altman analysis plot (full lines: absolute biases, dotted lines: 95% limits of agreement) (**a**) of cardiac output measurements as acquired with compressed sensing real‐time phase contrast MRI (CS RT) using RT cine with arrythmia rejection as a reference at the ascending aorta (AAo) and pulmonary artery (MPA) of patients with cardiac arrhythmia. Panel (**b**) shows an example of real‐time flow curves in the aorta and pulmonary artery of one patient with arrhythmia. Ten patients with flow measurements in Aao, four of whom additionally had flow measurements in the MPA. Red: data acquired with CS RT, *R* = 7.9, SVE, blue: data acquired with CS RT, *R* = 13.3. AAo = ascending aorta; MPA = pulmonary artery; CS = compressed sensing; RT = real‐time; SVE = shared velocity encoding.

In patients with arrhythmia, aortic flow was acquired in all (N = 10), and pulmonary flow was additionally acquired in four. Bias from all 14 measurements was −0.4 ± 0.6 L/min (−8 ± 12%, *P* = 0.05) from CS RT, *R* = 7.9, SVE and −0.4 ± 0.6 L/min (−8 ± 11%, *P* = 0.03) for CS RT *R* = 13.3 with RT cine sequence with arrhythmia rejection as reference (Table 3 line marked with “arrhythmia”; Fig. 7a). Examples of non‐periodic flow curves in the AAo and MPA of a patient with arrhythmia are shown in Fig. 7b.

#### EVALUATION OF RT CINE

The validation of RT cine with arrhythmia rejection in sinus rhythm showed left ventricular cardiac output bias of 0.05 ± 0.5 L/min (−0.7 ± 9%) compared to the reference PC gated acquisition.

#### INTEROBSERVER VARIABILITY

Phantom cardiac output differed by −0.1 ± 0.2 L/min (−1 ± 2%) for the gated PC, −0.3 ± 0.3 L/min (−4 ± 4%) for CS RT, *R* = 7.9, SVE and −0.2 ± 0.3 L/min (−3 ± 5%) for CS RT, *R* = 13.3 between observers. In patients in sinus rhythm and no valve disease these differences were 0.0 ± 0.1 L/min (0.2 ± 2%), 0.1 ± 0.3 L/min (2 ± 5%) and −0.0 ± 0.3 L/min (−0.1 ± 4%) for each protocol respectively.

Moreover, the differences in patients in sinus rhythm and suspected aortic regurgitation were 0.1 ± 0.2 L/min (2 ± 4%) for the gated PC, −0.1 ± 0.4 L/min (−2 ± 8%) for the CS RT, *R* = 7.9, SVE protocol and −0.2 ± 0.4 L/min (−3 ± 8%) for CS RT *R* = 13.3. Regurgitation fraction quantification differed by −0.1 ± 0.6% (−2 ± 6%), 0.2 ± 1.2% (2 ± 8%) and −0.0 ± 1.7% (5 ± 17%) for each protocol respectively.

In patients with arrhythmia, interobserver variability was −0.4 ± 0.6 L/min (−9 ± 12%) for CS RT and −0.5 ± 0.6 (−1 ± 9%), 0.2 ± 0.3 L/min (3 ± 6%) for RT cine cardiac output.

RT cine cardiac output variability in sinus rhythm patients was 0.2 ± 0.3 L/min (3 ± 6%).

In in vivo studies, the different CS RT protocols resulted in biases that did not differ more than 7.5% across parameters (Table 3).

## Discussion

This study validated a two‐dimensional RT PC research sequence with online CS reconstruction against established gated PC flow MRI in 1) a flow phantom and 2) patients in sinus rhythm with and without aortic regurgitation, and against RT cine in 3) patients with arrhythmia. Phantom validation showed high accuracy in cardiac output but underestimation of peak flow rate in imaging planes simulating the ascending aorta and the pulmonary artery, especially at higher heart rates. In sinus rhythm patients, high accuracy for cardiac output and regurgitation was observed, but underestimation of peak flow rate. In arrhythmia patients, the CS RT method provided accurate cardiac output quantification.

### Accuracy of CS RT Flow

#### IN VITRO

Cardiac output quantification with the CS RT method was accurate. Bias was low for all experiments, within expected error margins for PC MRI.^4^ Discussion on the phantom setup can be found in the Supporting Information.

Peak flow rate was underestimated more for higher heart rates. Underestimation might be due to low temporal resolution in relation to the short pulsation cycle and temporal regularization enforced by the CS algorithm.^24^ This is supported by results from Kowalik et al^17^ and Yang et al^25^ who demonstrated reduced underestimation of peak flow by improving acquisition or reconstruction, respectively. Future work could investigate such developments to reduce underestimation of peak flow.

#### IN VIVO

The CS RT sequence demonstrated accuracy and consistency in a clinical setting. However, peak flow measurements showed higher underestimation than in the phantom. This may be due to flow profile differences. The phantom systolic flow had a wider profile compared to the narrower and steeper systolic flow observed in vivo, which could be more susceptible to regularization effects.

The CS RT regurgitation fraction was underestimated. This could be attributed to the combination of low temporal resolution in relation to the duration of backward flow and CS temporal regularization. The underestimation led to change in severity class categorization in 2 of 10 patients compared to the reference.^23^ Further investigation is needed to fully understand the clinical utility of CS RT protocols in aortic regurgitation. Within the cohort of patients with suspected aortic regurgitation, some were found to have a low regurgitation fraction. Underestimation by CS RT in these patients resulted in high relative errors which influenced the overall relative bias. However, we do not consider this large relative bias representative of CS RT performance since these errors occurred in regurgitation fractions <15%, which is a clinically non‐important range.^23^

CS RT flow captured non‐periodic flow in arrhythmia patients, providing accurate online calculation of cardiac output from a 10‐second scan. This may be relevant for imaging in patients where gated methods fail, such as in atrial fibrillation or other arrhythmias, or in presence of severe motion such as exercise MRI.^26^

Peak flow rate quantification in patients did not show a dependency on heart rate, in contrast to phantom data. This suggests that the proposed method maintains peak flow rate bias <20% over the range of heart rates seen in the patients, while the underestimation increases in the higher heart rates seen in the phantom. There was no effect of heart rate on cardiac output suggesting that CS RT maintains similar accuracy in vivo and phantom.

Results from the different acceleration settings were similar. This shows that an adequate temporal resolution can be achieved either with a high CS acceleration factor, or with lower CS acceleration factor and SVE. SVE may therefore be useful for future flow sequence development.

### Relation to Previous Studies

Recently, Xiong et al^19^ validated the same sequence as presented here with CS acceleration *R* = 7.9 and SVE, in a phantom and healthy volunteers. They demonstrated that CS and SVE can be successfully combined to increase reconstructed temporal resolution without compromising accuracy of cardiac output. Our study extended the applicability of the sequence to a clinical setting, demonstrating feasibility in cardiac patients with or without valvular disease or arrhythmia. Our patient results showed similar bias as Xiong et al in healthy subjects,^19^ suggesting that the method's accuracy was not affected by more challenging imaging conditions seen in patients such as heavy breathing and bulk motion. Furthermore, Haji‐Valizadeh et al^14^ developed an RT flow sequence with higher acceleration factor and offline CS reconstruction, with validation in a pediatric population. Our online results are in concordance with previously reported data from offline reconstruction for cardiac output, peak flow rate and regurgitation fraction.^17^

Underestimation of peak flow rate or velocity can be an issue in, for example, stenotic valvular disease and development to circumvent this issue is desirable. Sun et al^12^ lowered peak velocity bias from RT with low‐rank reconstruction. Haji‐Valizadeh et al^18^ used deep learning for artifact filtering of undersampled datasets. However, these approaches include offline image reconstruction, hindering clinical use. An online approach by Joseph et al^16^ demonstrated peak velocities that were sometimes overestimated with undersampled RT compared to standard gated PC, hinting that RT might be more accurate than the reference. This highlights the issue of evaluation of peak flow rates or velocities with MRI where getting a true reference is challenging.

### Clinical Implications

This study demonstrated that the CS RT flow sequences investigated can provide accurate cardiac output in arrhythmia patients, where established methods are limited. Furthermore, CS RT can be of use when clinical protocols need to be heavily optimized for scan time. This applies to patients that find long scanning times challenging (eg patients with orthopnea or pediatric patients) or patients under anesthesia where scan time minimization reduces risk (eg pediatric patients) or patients where flow imaging is required in multiple planes (eg congenital heart disease). Rapid scan times can also be achieved with gated PC sequences,^27^ however, in contrast with RT, breath‐holding is required. CS RT could also be used during physical exercise where a reliable ECG gating signal is not always available.

### Limitations

The phantom validation was limited to periodic forward flow experiments and was therefore restricted from simulating valvular regurgitation.

The CS spatial and temporal regularizations suppress small details in the image and artificially reduce noise. Therefore, velocity‐to‐noise ratio (VNR) measurements in CS images were not possible, since the ratios were artificially inflated and not directly comparable with reference.

A relatively low number of patients was included in each cohort and further investigation of the method is required to draw firm conclusions. Furthermore, only four patients had CS RT data in both AAo and MPA. Therefore, a systematic investigation of flow consistency (*Q*_*p*_/*Q*_*s*_) was not possible.

This study did not investigate whether longer scan windows would give a smaller difference between gated and CS RT acquisitions. Yet, with more than five heartbeats included in the analysis, we anticipate that this should suffice for a representative cardiac output computation.

Finally, our CS RT validation in arrhythmia was limited to cardiac output quantification due to lack of a reference method for peak flow rates. While ultrasound is first‐line assessment of flow rates and velocities clinically, it was not included as a reference since ultrasound is also challenged in the assessment in arrhythmic patients^28^, ^29^, ^30^ and measurements are not comparable with MRI.^29^

## Conclusion

Real‐time flow imaging with fast online compressed sensing image reconstruction (CS RT flow) has potential to be used for fast, accurate flow assessment in the presence of aortic regurgitation or arrhythmia.

## Supporting information


**Data S1:** Supporting Information.
