# Supplementary material for: Evaluation of Real‐Time Cardiovascular Flow MRI Using Compressed Sensing in a Phantom and in Patients With Valvular Disease or Arrhythmia
Source: J Magn Reson Imaging. 2025 Jan 20;62(2):417–29. doi: 10.1002/jmri.29702 (PMC12276637; doi:10.1002/jmri.29702)
Supplement: Supplementary file 1 — Data S1: Supporting Information. [file JMRI-62-417-s001.pdf]

Supplementary Information

Evaluation of real-time cardiovascular flow MRI using compressed sensing in a phantom and in patients with valvular disease or arrhythmia

Tania Lala[1,2], MS, Lea Christierson[2,3], MS, Petter Frieberg [1], MD, PhD, Daniel Giese[4], PhD, Peter Kellman[5], PhD, Nina Hakacova[3], MD, PhD, Pia Sjöberg[1], MD, PhD, Ellen Ostenfeld[1], MD, PhD, Johannes Töger[1,2], PhD.

[1]*Clinical Physiology, Department of Clinical Sciences Lund, Lund University, Skåne University Hospital, Lund, Sweden,*

[2]*Biomedical Engineering, Lund University, Lund, Sweden,*

[3]*Pediatric Heart Centre, Department of Clinical Sciences Lund, Lund University, Skåne University Hospital, Lund, Sweden,*

[4]*Magnetic Resonance, Siemens Healthineers GmbH, Erlangen, Germany,*

[5]*National Heart, Lung, and Blood Institute, Bethesda, MD, United States*

Methods, In vitro experiments, Description of flow phantom

Flow was produced by a pulsatile pumping system, with the pump driven by a motor-powered actuator, and a reservoir of tap water. The pump was made in house entirely in MR-safe materials. The piston was driven by a Myostat RD 55 12 150 actuator powered by a Cool Muscle 2 CM2 X 5620A servo motor (Myostat Motion Control Inc, Newmarket, Canada). The actuator motor unit was controlled via a USB connection with a laptop by the corresponding software Control Room (Myostat Motion Control Inc., Newmarket, ON Canada), all placed outside the 2 mT safety line in the MRI scanner room. The pumping system was modified from a previously used design (1) to enable higher flow and pulsation frequencies.

The start and end positions of the piston as well as the maximum velocity and acceleration were controlled and specified for each pump cycle. In the in vitro experiments, the parameters were set to be the same for each cycle generating periodic flow. Cardiac gating was simulated by a triggering signal generated by the motor actuator system for each pumping cycle.

Results, In vitro, CS RT in the phantom, Background phase analysis

Background phase analysis showed mean velocity in the phantom AAo of -1.6±0.1 cm/s for the gated sequence, -0.5± 0.7 cm/s for CS RT, R=7.9, SVE and 0.2± 0.2 cm/s for CS RT, R=13.3. In the phantom MPA the mean velocity was -1.5±0.2 cm/s for the gated sequence, -1.0±0.6 cm/s for CS RT, R=7.9, SVE and -0.3±0.7 cm/s for CS RT, R=13.3.

Results, In vivo, CS RT in sinus rhythm and no valvular disease, Background phase analysis

Background phase analysis showed mean velocity in the AAo of -1.0±1.0 cm/s for the gated sequence, -0.7±1.1 cm/s for CS RT, R=7.9, SVE and 0.4±1.5 cm/s for CS RT, R=13.3. In the MPA the mean velocity was -0.4±0.2 cm/s for the gated sequence, 2.0±2.0 cm/s for CS RT, R=7.9, SVE and 2.3±1.5 cm/s for CS RT, R=13.3. Mann Whitney tests showed that background velocity was statistically significantly lower in CS RT protocols (R=7.9, SVE: $p=0.025$, R=13.3: $p< 0.0001$).

Discussion, Phantom setup

Cardiac output in the phantom was stable throughout the MRI protocol. The reference acquisition overestimated true flow in the AAo plane and underestimated true flow in the MPA plane, by a low bias which was in line with previous knowledge on the bias introduced by the number of pixels/diameters in the experiments. A systematic underestimation in the MPA plane may be due to partial volume error introduced by the flow profile that was observed in the angled part of the tube with high velocities at the bottom side of the tube, i.e. high velocity pixels at the edge of the region of interest (2). However, this is true for both the reference and the CS RT acquisitions and therefore any bias introduced due to this should not differ notably between the two methods. Furthermore, our phantom included stationary water close to the tube which allowed for investigation of phase background effects for different flow sequences.

References

1. Töger J, Andersen M, Haglund O, Kylkilahti TM, Lundgaard I, Markenroth Bloch K. Real-time imaging of respiratory effects on cerebrospinal fluid flow in small diameter passageways. Magnetic Resonance in Med. 2022 Aug;88(2):770–86.

2. Wolf RL, Ehman RL, Riederer SJ, Rossman PJ. Analysis of systematic and random error in MR volumetric flow measurements. Magn Reson Med. 1993 Jul;30(1):82–91.
